# Infectious Disease Hospitals in London

**Published:** 1893-12-16

**Authors:** 


### THE INSTITUTIONAL WORKSHOP.

### HOSPITALS OF THE WORLD.*

### VII.—INFECTIOUS DISEASE HOSPITALS IN LONDON.

ONE of the dangers which seemed to Queen Elizabeth so formidable in the rapid growth of London was that to which the inhabitants would be exposed from epidemic disease. That her fears were well-founded appears from the history of the metropolis since her day, though the ravages of diseases which have again and again decimated the population are held of little account by historians in comparison with the squabbles of parliamentary debaters. At the present moment there is probably no city in the world better equipped for the double work which infectious complaints entail upon the community, viz., the relief of the poorer patients unable to procure skilled attendance, and the isolation of others who become a danger to their neighbours when nursed in their own homes. These two functions, of which the first was for many years the only one recognised by the authorities, tend to become merged in one, as the value attaching to the prevention of disease becomes more fully understood.

The sole provision formerly made for those suffering from epidemic disease was the sick wards of workhouses. At the commencement of this century by voluntary effort the London Fever Hospital (known as the House of Recovery), and the London Small-pox Hospital were built, to which patients were admitted on the order of the relieving officer. But it was not until 1886 that any movement was made by the legislature. The Sanitary Act of that year provided that the sewer authority, or in the metropolis the nuisance authority, might provide for the use of the inhabitants within its districts hospitals or temporary places for the reception of the sick. The following year the Metropolitan Poor Act, introduced by Mr. Gathorne Hardy, called into being the Metropolitan Asylums Board; fever and small-pox hospitals were built at Hampstead (a temporary building), Homerton, and Stockwell; and a serious small-pox epidemic (1870) tested to the utmost the new attempt of the State to battle with infection. Two points became clear at the close of the epidemic. First, that the applications for admission to these new hospitals came from persons in almost all ranks of life—fully 90 per cent. were above the pauper class. Secondly, that a grave danger was forced upon the inhabitants of the districts immediately surrounding the hospitals, where it was unmistakeably evident that there was an excessive incidence of small-pox. Subsequent investigations showed that the percentage of houses invaded in the neighbourhood of the hospital became gradually smaller as the distance of the houses from the hospital increased. This gradation was very exact and very constant. The injury caused to the districts in question did not cease with the cessation of the epidemic. Houses were abandoned, the value of land decreased, and the neighbourhoods were left to a poor population tempted by cheap rents. Before these facts were generally established, further fever and small-pox hospitals were built at Fulham, Deptford, and Poplar. But in 1882 the difficulties of the managers, in face of the legal difficulties which beset them, became so serious that a Royal Commission was appointed to consider the whole question. This Commission, after a full investigation, made the following recommendations: That small-pox patients should be sent down the river to hospitals in isolated situations on its banks, or to floating hospitals on the river itself; that for those too ill to be moved from their immediate neighbourhood small-pox hospitals for some thirty or forty patients should be erected within the precincts of the fever hospitals; London to be divided into districts so that no hospital should receive patients from any but its own neighbourhood. That these hospitals should be so constructed as to minimise the risk of spreading infection, evidence being before the Commission to show that hospital wards might be constructed so as to enable the air after it has passed through the hospital to be subjected to high temperature or some other means of destroying whatever dangerous properties it may possess before it is discharged. The Commission held that the amount of accommodation for infectious disease necessary for London was 3,000 beds for fever patients, and from 2,100 to 2,700 beds for small-pox patients. As regards administration, the Commissioners recommended that the provision of accommodation for infectious patients should be entirely disconnected from the poor law administration; that all patients should be treated alike, but that separate pay wards should be provided for those who desired it. They also strongly urged a system of compulsory notification of infectious disease, the immediate outcome of which recommendation was the Infectious Disease (Notification) Act, 1889.

The hospital sites at Darenth, in Kent, and Winchmore Hill, four miles to the North of London, were purchased as the result of these recommendations; the hospital ships "Atlas" and "Endymion" were removed to Long Reach, near Dartford; and an admirable ambulance system was organised. This ambulance system, playing so important a part in the London defences against infection, is twofold—for land and river service. There are three stations connected with the land ambulance service, situated respectively near the Western Hospital at Fulham, the Eastern Hospital at Homerton, and the South-Eastern Hospital at Deptford. Each possesses coach-house, stables, disinfecting-rooms, laundry, kitchen, dormitories, and lavatories for the staff, which consists of a superintendent, drivers, helpers, nurses, and a telephone clerk, for every ambulance station is in telephonic communication with the central office in Norfolk Street. Well-arranged ambulance carriages are ready at any moment to proceed for the removal of a patient, accompanied by two men and a nurse. The ambulance contains a bed upon which the patient can lie down, and, as a rule, is furnished for one case only. It is well ventilated, and constructed of polished wood, inside and out, admitting of ready disinfection. Within three minutes of a summons being received from the central office for the removal of a patient, the ambulance has already started on its journey.

For the river service there are three wharves: the west, on the northern bank of the river at Fulham; the south, on the southern bank at Rotherhithe; and the north, on the northern bank of the river at Poplar. The removal of patients is effected in three steamers, the "Red Cross," the "Maltese Cross," and the "Albert Victor," each fitted with ample separate accommodation for patients of both sexes, and having on board all the appliances for the treatment and feeding of the sick. The arrangements in these steamers are so complete that from the moment the patient is put to bed on board, his hospital treatment may be said to begin.

The value of the Asylums Board Hospitals has been increased by the regulation now in force that the certificate of any legally-qualified practitioner suffices as an order for admission instead of that of the relieving officer. The Board has also undertaken to remove persons suffering from infectious disease to other hospitals than their own, thus avoiding the necessity for a double ambulance system in the metropolis. They have, further, made arrangements to admit students (under proper precautions) to the practice of their hospitals, which have thus become schools of medicine.

The Infectious Disease (Prevention) Act, 1890, has given

* "Hospitals and Asylums of the World," 4 vols., and Portfolio of Plans. (Messrs. Churchill and the Scientific Press.)

to the authorities who adopt it enlarged powers as regards disinfection of clothes, dwellings, &c., and the supervision of suspected milk services, dairies, &c., and thus materially strengthened the forces at work against infection.

The extent to which these hospitals have gained the confidence of the poor must be reckoned as not the least admirable part of the work which they are achieving. Fifty years ago it may be safely conjectured that an edict for the compulsory removal of fever and small-pox patients from their homes would have met with the most determined resistance.

Yet now, even among the most ignorant and suspicious of the London poor, these hospitals are mentioned with gratitude. The little patients are entrusted to them by the parents with unbounded confidence, and stories of kindness bestowed and real personal sympathy exhibited are related no less cordially when the disease has proved fatal than when the patient has been restored in full health to his friends.

